# Dr. Fisher’s Case of Tumour Developed in the Midst of the Cauda Equina

**Published:** 1842-06-04

**Authors:** W. W. Fisher

**Affiliations:** Downing Professor of Physic, Cambridge


*Case of Tumour developed in the midst of the Cauda Equina.* By W. W. Fisher, M. D., Downing Professor of Physic, Cambridge.—The case I am about to communicate came under my observation in January, 1840. The name of the individual affected was Taylor; he was thirty-eight years of age, was a tailor by trade, and had been intemperate in his habits for some time previous to the commencement of his illness. He had about three years before (i. e. in 1837,) injured, whilst riding, the lower part of the loins by the back part of the saddle, and from that period he began to suffer from pain in the lumbar and sacral regions, which was attributed to rheumatism; the pain gradually became more violent, and extended down the legs, which began to swell. He was obliged to give up work in June, 1839, and became bed-ridden in the September of the same year; he could not, however, lie down, but rested on his hands and knees, in which position I found him when I first saw him. He was then unable to move either his loins or lower extremities; but he had the free use of neck, shoulders, and arms. The pain, which had formerly been chiefly confined to the region of the sacrum, was now more particularly felt across the seat, extending from one ischium to the other. There was great numbness throughout the lower extremities; and although no sensation was in the left leg or toes by touch, nevertheless he complained strongly of a feeling of heat in the parts. There was some degree of feeling, on touch, left in the right leg. The legs were very œdematous; there were large ulcerations on those parts of the knees on which he rested, yet he did not experience pain from them. He was generally sleepless, but did not suffer from headache; his breathing was easy, his pulse undisturbed, and his appetite good. He had difficulty in making water; and his bowels were generally confined, and at times so obstinately constipated as to resist the action of cathartics and purgative injections. An issue had been placed on the region of the sacrum, the discharge from which was thin; this was rendered of a more purulent character by the use of iron, from which he seemed to derive more benefit than from any other medicament, especially as regarded the making of water.

His death took place in May last. He had been able, about a fortnight before it occurred, to place himself on his back, after which the œdema of the legs subsided; there was, however, incontinence of the urine, which was at times mixed with blood. He slept better, and was even able to assist in making a waistcoat about a week before he died.

*Post-mortem.*—Owing to peculiar circumstances, I was only able to examine the back; imperfect, however, as the investigation was, it furnished me with a morbid product sufficient to account for the peculiarily of the symptoms, and the situation and structure of which must be a matter of interest in pathology. The sacrum seemed more protuberant than usual; this appearance, however, arose from the loins being more depressed. The arches of the dorsal and lumbar vertebræ and the posterior wall of the sacrum were removed; the laminæ of the lumbar vertebræ, as well as their bodies, were partially affected with caries.

Viewed posteriorly, the dura mater appeared to be in its natural state until it reached the extremity of the spinal cord; but from that point to the end of the sacrum it was wanting, so that the mass of tumours was exposed to view. The growth extended more towards the left than the right side of that portion of the spinal canal in which it was situated. The spinal cord was cut across, about the middle of the back, and inferior portion of it was removed; nearly the whole of the diseased mass came along with it. The dura mater was laid open posteriorly. The cord appeared to be quite sound throughout. The diseased mass had a lobulated form, and was involved in the cauda equina; and although it was traversed by a few of the nerves, nevertheless the greater portion of the latter could be detached from it.

It was difficult to determine the seat of the tumour when examined posteriorly; but anteriorly the dura mater was sound throughout; and the arachnoid membrane, especially at the upper portion of the tumour, could be traced intact between the latter and the dura mater. Here and there processes were observed to pass from the arachnoid to the diseased structure, but they were similar to those met with between the arachnoid and the pia mater in their natural state. The tumour, or rather mass of tumours, on which a great number of vessels were spread, was, as I observed before, surrounded on all sides by the roots of the nerves forming the cauda equina. The lower portion of this morbid growth had the form of a tubercle. It presented several traces of vascularity in the centre, and had a scirrhous appearance; I could not however make anything out satisfactory with regard to its minute structure. The upper portions of the tumour were softer, and were involved in a fine glistening covering; sections of several portions of them showed them to be composed of a grey, semi-transparent, jelly-like substance, infiltrated amidst reticulated tissue, and marked with sanguineous striæ, several of which appeared like true vessels.

I had not an opportunity of having any part of this morbid growth analysed. I plunged a portion of it in pure alcohol, and as it retained its semi-transparency, I concluded it was not albuminous.

Notwithstanding the researches I have made, I have not yet been able to meet with any case on record similar to the one just described. Amongst the many points of interest which the tumors offer—as, for instance, the region they occupy, the peculiarity of their structure, the symptoms they gave rise to—I shall on the present occasion allude more particularly to one, that is to say, the tissue in which they were developed. There can be little doubt that the disease was seated in the pia mater, a vascular web in which morbid products are more frequently formed than in either of the other membranes of the brain. I have observed in a diseased state of the pyloric extremity of the stomach, the walls of which were greatly thickened, a morbid product, which was seated between the serous and muscular layers, and of which the minute structure was similar to that of the section of the tumour in question.

There exists a great analogy between the pia mater of the brain and spinal cord and the subserous tissue of other organs; and, indeed, where the former is united with the arachnoid, the analogy becomes complete. Observation has led me to consider the subserous cellular tissue as being more frequently than any other tissue of the human frame the seat of morbid products. I shall not however enter at length into this subject on the present occasion, but shall defer till another the statement of the facts on which this opinion is grounded.—*Transactions of the Provincial Association*, Vol. X.

